# Draft Genome Sequence of Burkholderia vietnamiensis Strain RS1, a Nitrogen-Fixing Endophyte Isolated from Sweet Potato

**DOI:** 10.1128/MRA.00820-18

**Published:** 2018-07-26

**Authors:** Rina Shinjo, Kazuma Uesaka, Kunio Ihara, Shunsuke Sakazaki, Katsuya Yano, Motohiko Kondo, Aiko Tanaka

**Affiliations:** a Graduate School of Bioagricultural Sciences, Nagoya University, Chikusa, Nagoya, Japan; b Center for Gene Research, Nagoya University, Chikusa, Nagoya, Japan; University of Arizona

## Abstract

Burkholderia vietnamiensis strain RS1 is an endophytic bacterium with nitrogen-fixing ability that was isolated from tuberous roots of sweet potato. Here, we present its draft genome of 6,542,727 bases that contains a cluster of genes associated with nitrogen fixation. This genome sequence will provide important insights into the plant growth-promoting potential of endophytic bacteria.

## Abstract (precis)

Burkholderia vietnamiensis strain RS1 is an endophytic bacterium with nitrogen-fixing ability that was isolated from tuberous roots of sweet potato. Here, we present its draft genome of 6,542,727 bases that contains a cluster of genes associated with nitrogen fixation.

## ANNOUNCEMENT

Sweet potato (Ipomoea batatas) is a staple root crop in Asia and Africa and can grow well in infertile nitrogen-poor soil (1). Endophytic nitrogen fixation by microorganisms has been suggested to contribute to nitrogen acquisition from the atmosphere in sweet potato (2, 3). However, there is little information about the genetic and physiological characteristics of endophytic bacteria of sweet potato. Understanding these characteristics would elucidate the molecular mechanisms of plant-diazotroph symbiosis and thereby promote effective utilization of nitrogen-fixing bacteria.

Bacterial strains were isolated from tuberous roots of I. batatas cv. Beniazuma, grown in a pot placed in an experimental field at Nagoya University, Nagoya, Aichi, Japan. The tuberous roots were cut into pieces (weighing approximately 5 g [fresh weight] each) and washed under running tap water for 30 min. The washed tissues were surface sterilized by soaking in 70% ethanol for 1 min and then in 0.3% NaOCl (in 0.002% Tween 20) for 2 min. After surface sterilization, the tuberous root tissues were rinsed three times with sterile water, ground in a sterilized mortar, and suspended in 5 ml of 0.3 M phosphate buffer containing 0.2 M sucrose. A 50-μl aliquot of ground material was plated onto a yeast mannitol agar medium (4) and incubated for 5 days at 28°C under aerobic conditions. The sequenced bacterium RS1, which was selected as an *nifH*-containing strain, was identified as Burkholderia vietnamiensis based on partial sequencing of 16S rRNA. B. vietnamiensis is a diazotrophic bacterium belonging to the Burkholderia cepacia complex. Other strains of B. vietnamiensis have been isolated from soil or roots of rice, sugarcane, and nipa palm (5–7).

The genomic DNA of B. vietnamiensis RS1 was extracted using a genomic DNA purification kit (Promega, USA). Paired-end DNA libraries were prepared for sequencing with the MiSeq platform (Illumina, USA). A total of 641,426 high-quality paired-end reads were generated. *De novo* assembly was performed using the SPAdes genome assembler version 3.9.0 (8) with three options, “-k auto,” “-careful,” and “-cov-cutoff 10.0," which yielded 70 contigs. The draft genome of B. vietnamiensis strain RS1 consists of 6,542,727 bases with a G+C content of 67.6%. Annotation of the contigs was performed using the DFAST version 1.0.0 pipeline (9), which identified 5,797 putative coding sequences.

The genome annotation confirmed the presence of the *nif* gene locus for nitrogen fixation. The *nif* gene cluster, which is 46.4 kb in length, is composed of 61 open reading frames, including *nifQXNEKDHOTSZZBAUVPW* and *nif-*related genes (*modACE*, *fdxBN*, and *fixAB*). In addition, several genes related to plant growth promotion, such as siderophore production and 1-aminocyclopropane-1-carboxylic acid degradation, have been identified. A comparison of the RS1 genome with other B. vietnamiensis genomes (10, 11) revealed high similarity to B. vietnamiensis strain LMG 10929 (=TVV75), with 98.7% average nucleotide identity (12).

### Data availability.

The genome sequence of B. vietnamiensis strain RS1 was deposited in DDBJ/EMBL/GenBank under the accession no. BGKC01000001 to BGKC01000077.
